# Frequency of Physical Activity Done with a Companion: Changes Over Seven Years in Adults Aged 60+ Living in an Australian Capital City

**DOI:** 10.1177/08982643231158424

**Published:** 2023-02-28

**Authors:** Genevieve S. E. Smith, Wendy Moyle, Nicola W. Burton

**Affiliations:** 1 School of Applied Psychology, 170572 Griffith University, Brisbane, QLD, Australia; 2 Menzies Health Institute Queensland, 300151 Griffith University, Brisbane, QLD, Australia; 3 Centre for Mental Health, Griffith University, Brisbane, QLD, Australia, Brisbane, QLD, Australia; 4 School of Nursing and Midwifery, 539324 Griffith University, Brisbane, QLD, Australia

**Keywords:** older adult, active aging, companionship, physical activity, social support

## Abstract

**Objectives:**This study examined how often adults 60+ years were physically active with a partner, close family, friends, and neighbors, over 7 years. **Methods:** Data from 2062 adults living in an Australian capital city were collected using a mail survey at four time points and analyzed using multinomial logistic regression. **Results:** A partner was the most frequent companion at all time points. From baseline to 7 years, the greatest decline was activity with family 1–4x/month (.79 [.64–.98]) and ≥5x/month (.54 [.36–.80]). There were also decreases in activity 1–4x/month with a partner (OR = .75, [.62–.92]), friends (.55 [.44–.68]), and neighbors (.79 [.64–.98]). Physical activity with friends or neighbors ≥5x/month did not decline. **Discussion:** Findings extend understanding of physical activity and activity companions among older adults. More research is needed to understand factors contributing to changes in activity done with companions.

Physical activity is one of older adults’ leading determinants of health and wellbeing (Bauman et al., 2016). Older adults who engage in sufficient physical activity have a slower rate of age-related physical decline; and a reduced risk of chronic conditions, cognitive decline, and poor psychological health than those who are inactive (Bauman et al., 2016; Hagen et al., 2012). In addition, physical activity can increase positive psychological wellbeing (Black et al., 2015), life satisfaction (Ku et al., 2015), health-related quality of life (Halaweh et al., 2015), confidence, mastery, and self-esteem (McAuley et al., 2000). Older adults who are physically active use fewer primary health care services than inactive older adults and use those services less frequently (Sari, 2011). It has been estimated that 20%–60% of older adults are meeting the World Health Organization (WHO)’s guidelines of a minimum of 150 min/week of moderate to vigorous physical activity (Sun et al., 2013). However, this has declined during the COVID-19 pandemic (Cunningham & O’ Sullivan, 2020). Accordingly, more research is needed to understand the factors that positively influence physical activity among older adults.

Social support can potentially influence physical activity uptake and maintenance in older adults. It is a multidimensional construct broadly defined as emotional and practical assistance underpinning good social relations (WHO, 2003) and has several subtypes including companionship (Martin et al., 2013; Scarapicchia et al., 2017; Stapleton et al., 2015). Companionship can positively influence physical activity through encouragement, connectedness, and social norms. Encouragement includes praise and confidence for participation, connectedness includes friendships and networks that facilitate participation, and social norms set the standards of behavior. These processes may increase self-efficacy and motivation for physical activity (Thoits, 2011). Companionship can come from various sources, including partners, family, friends/peers, colleagues, professionals, and community groups.

A cross-sectional population survey from Brazil reported that companionship for physical activity was one of the strongest social support subtypes correlated with physical activity in older adults (da Silva et al., 2012). Salvador et al. (2009) reported that older men invited by their friends to participate in physical activity were 3 times more likely to reach recommended physical activity levels than those not invited. Böhm et al. (2016) reported that older adults who had the company of family and friends during physical activity were more than twice as likely to reach physical activity guidelines than those who did not have company (50% vs. 20%). A meta-analysis on physical activity preferences found that as older adults age, their preference for exercising with others may increase (Amireault et al., 2019)

In addition to enabling participation, physical activity companionship can improve psychological and physical health. Cross-sectional research by Seino et al. (2019) found that compared to those who exercised alone, those who exercised with companions were 32% more likely to reach sufficient physical activity and 45% more likely to have good mental health. A longitudinal study over 4 months by Kritz et al. (2021) found that compared to those who walked alone, those who regularly walked with companions had a significantly greater increase in their physical activity performance (pace and distance) and showed greater improvements in self-efficacy, autonomous motivation, fat loss and functional capacity.

A cross-sectional study by Böhm et al. (2016) found that 13% of older adults walked with family members, 7.9% walked with friends, and only 2.4%–3.8% had company from family and friends for moderate to vigorous activity. Limited research however has explored if/how physical activity done with companions changes over time in older adults. Older adults may be vulnerable to general companionship disruptions due to life events such as retirement, change in other roles, loss of significant others and friends, and reduced physical functioning (Charles & Carstensen, 2010). For example, Bruine de Bruin et al. (2020) found a 50% reduction in social network size between 50–59 and 60–69 years. The current study aimed to explore how often adults 60+ years were physically active with companions and if this changed over 7 years.

## Method

### Study Design

This study used prospective data sourced from HABITAT, a multilevel study of health and recreation in people aged 40–65 years at baseline in Brisbane, the capital city of Queensland in Australia. A brief overview of the HABITAT study is provided below, with more details available elsewhere (Burton et al., 2009).

### Ethics

HABITAT was initially awarded ethical clearance by The University Human Research Ethics Committee at the Queensland University of Technology (ID3967H). Survey completion and return were taken as informed consent.

### Participants and Sampling

Participants for the current study were those HABITAT participants aged 60+ years in 2009 (*N* = 2029) as this was the first year that physical activity done with companions was assessed. Participants in the overarching HABITAT study were recruited in 2007 using a two-stage sampling design with study areas selected first and then individuals. First, census collection districts (CCDs) were ranked into deciles using the Australian Bureau of Statistics (ABSs) index of relative socioeconomic disadvantage (IRSD). Then 20 CCDs were randomly selected from each decile to obtain socioeconomic diversity. Next, within each CCD, a random sample of people aged 40–65 (as the age group of interest) was identified using Australian electoral roll data from March 2007. Potentially eligible HABITAT participants (*n* = 17,000) were sent surveys in May 2007, and 11,035 responses were received from people aged 40–65 years at baseline (Burton et al., 2009).

### Procedure

Data were collected using a mail survey method by Dillman (2007). People initially received a personalized letter explaining the study purpose and the importance of the response. The mail survey was sent a week later with a reply-paid envelope for return. After 1 week, a thank you/reminder card was sent. Seven weeks after the initial mail-out, a personalized reminder letter and replacement survey, with a reply-paid envelope for return, was sent to non-respondents. At each wave, participants in the database were sent the survey, regardless of whether they had replied to the survey in previous years, except those who had actively withdrawn or were identified as having died. The current study used mail survey data from 2009, 2011, 2013, and 2016.

### Measures

#### Frequency of Physical Activity with Companions

The frequency of doing physical activity with companions was measured using 5-items from Giles-Corti and Donovan (2002). Participants were asked to rate the frequency in the past month they were physically active with each of the following 4 sources of companionship: spouse/partner, close family members, close friends, and neighbors. Responses were provided on a 7-point scale including 1 (Never), 2 (Once or twice), 3 (3–4 times), 4 (5–9 times), 5 (10 or more times), 6 (Not applicable), and 7 (Don’t know). Due to small cell sizes, responses were collapsed into 3 categories: 1 (Never), 2 (1–4x/month), and 3 (≥5x/month) to reflect doing physical activity with others never, at least monthly, or at least weekly. Cases with responses of 6 (Not applicable) and 7 (Don’t know) were excluded from the analysis.

#### Sociodemographic and Health Measures

The following constructs were assessed in the 2009 questionnaire and used for the baseline demographics of participants in the current study: gender (male/female), date of birth (day/month/year), country of birth (Australia/other specified), living arrangement (alone no children/single parent living with one or more children/single living with friends or relatives/couple living with no children/couple living with one or more child/other), and employment status (full-time work/part-time work/casual work/work without pay/home duties not looking for work/unemployed looking for work/retired/permanently unable to work/student/other). Self-rated health was measured using a single item asking, “In general, how would you describe your health?” on a Likert scale of 1-poor to 5-excellent. Physical activity was measured using items from the Active Australia questionnaire (AIHW, 2004) to assess time in the past week spent in walking, moderate and vigorous activity. These items were summed using the standard data management processes outlined in the survey manual and the Australian National Health Survey, with vigorous activity weighted by two to reflect greater intensity (ABS, 2021; AIHW, 2004). Physical activity was then categorized into meeting guidelines (≥150 weighted min/week) and not meeting guidelines (<150 weighted min/week), which reflects the WHO physical activity guidelines for older adults (WHO, 2020). Educational qualification (less than year 12/year 12/trade certificate, apprenticeship, diploma, certificate/bachelor’s degree/masters, or doctorate) was not measured in 2009. Therefore, 2007 data were used, assuming that changes to education status in this age group over the 2 years would be minor.

### Data Analysis Plan

Descriptive data were managed and analyzed using SPSS version 26. Multinomial logistic regression was used for all models with timepoint as a fixed effect, and the subject was included as a random effect to account for the probable non-independence of observations from the same participant. The covariates of gender, education, living situation, employment status, and self-rated health were added as fixed effects and tested to examine their association with companionship for physical activity due to prior research demonstrating an association with physical activity (Bauman et al., 2012). The Akaike information criterion (AIC) was used to select the most appropriate statistical model, where a lower AIC value indicates better fit quality relative to the model’s complexity (Bozdogan, 1987). The model with the lowest AIC was selected (see supplementary materials for models and AIC values). Four models, one per companionship type, are reported. Longitudinal analyses were performed in R statistical program version 4.1.2 (R Core Team, 2021). Models were analyzed using R packages Mclogit (Multinomial Logit Models, with or without Random Effects or Overdispersion) (Elff, 2021). This package estimates models with random effects (mixed conditional logit models) using maximum likelihood with a simple Laplace approximation. Figure 2 was created using the package GGplot2 (Wickham et al., 2021).

## Results

### Participant Characteristics

Participants were 2029 community-dwelling adults aged 60+ years at baseline (2009). Participants had a mean (standard deviation) age of 62.9 (2.1) years, and 61% were women. Just under a quarter (23%) of participants lived alone, approximately half (52%) had completed post-school qualifications, and 45% were currently in paid employment. Nearly two-thirds (63%) of participants were meeting physical activity guidelines (see Table 1).

**Table 1.** Summary Baseline Demographics of Current Study Participants by Gender (*n* = 2029).

Age (years)	Men *n* = 789	Women *n* = 1240	Total *n* = 2029
	M (SD)	M (SD)	M (SD)
	62.9 (2.2)	62.9 (2.1)	62.9 (2.1)
	*n* (%)	*n* (%)	*n* (%)
Country of birth			
Australia	565 (71.7)	950 (76.9)	1515 (74.9)
Other country	223 (28.3)	286 (23.1)	509 (25.1)
Education^a^			
Completed year 10 or less	196 (24.9)	531 (42.9)	727 (36.0)
Completed year 11 or 12	95 (12.2)	159 (12.8)	254 (12.5)
Certificate/Diploma	278 (35.4)	266 (21.5)	544 (26.9)
Bachelor’s degree or higher	216 (27.5)	282 (22.8)	498 (24.6)
Employment			
In paid workforce^b^	394 (54.3)	419 (38.1)	813 (44.6)
Not in paid workforce^c^	331 (45.7)	680 (61.9)	1011 (55.4)
Living arrangement			
Living alone	156 (20.5)	292 (24.6)	448 (23.0)
Living with others^d^	604 (79.5)	869 (75.4)	1500 (77.0)
Self-rated health			
Excellent/Very good	275 (36.2)	472 (39.0)	747 (37.9)
Good	330 (43.5)	487 (40.2)	817 (41.5)
Fair/Poor	154 (20.3)	252 (20.8)	406 (20.6)
Physical activity			
Meeting guidelines^e^	473 (63.9)	725 (62.7)	1198 (63.2)
Not meeting guidelines	267 (36.1)	432 (37.3)	669 (36.8)

*Note*. subcategories in the education, employment, and living arrangements variables were collapsed given small cell sizes. The measures section lists all possible response options. Education was measured in 2007.

^a^Measured in 2007.

^b^Includes full-time, part-time, and casual work status.

^c^Includes those working without pay, home duties, unemployment, students, and unspecified others.

^d^May include living with a partner, friend, family and/or children.

^e^Based on WHO physical activity guidelines of ≥150 weighted min/week (WHO, 2020).

**Figure 1.**
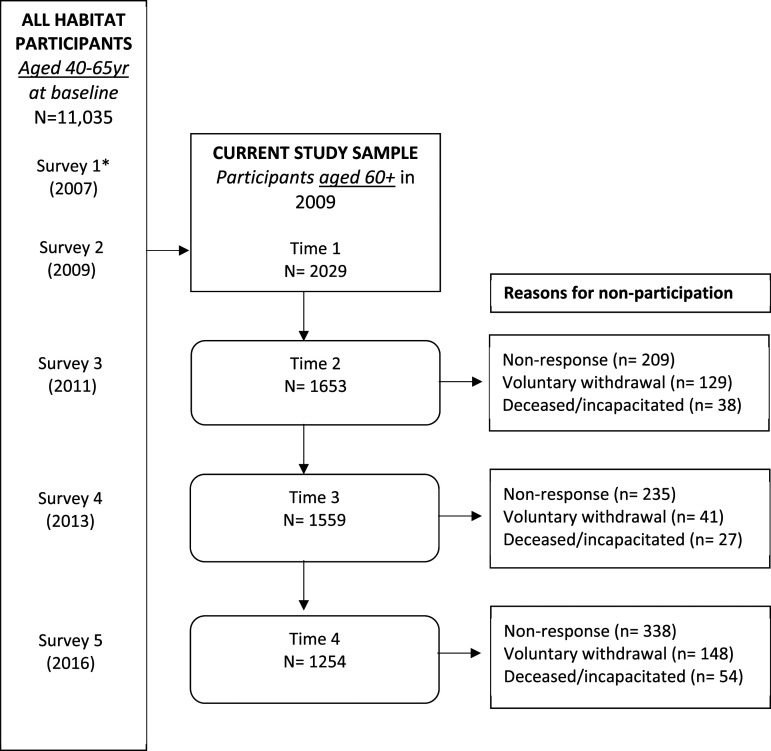
Flow of participants in the current study sample (aged 60+ in 2009). Note 1: Non-response is participants who did not return a survey or requested no further contact at the indicated timepoint but may have responded at subsequent time points. Note 2: *Survey 2 was where the current study sample was derived as this was the year that companionship began being assessed.

The flow of participants throughout the 4 time points of this study is presented in Figure 1. Over the 7 years of the study, 62% of participants were retained.

### Descriptive Results

**Figure 2.**
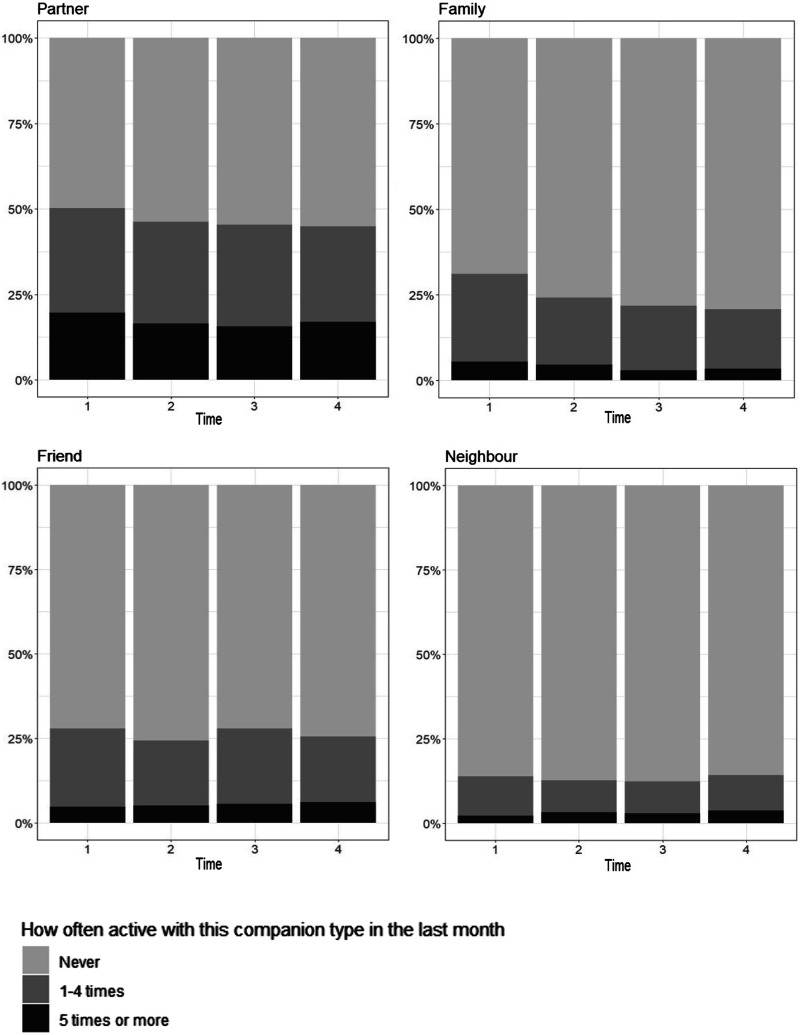
Frequency of physical activity in the past month with different companion types over 7 years.

Figure 2 summarizes the frequency of physical activity with each of the 4 companion types over time. Descriptive results indicated that at baseline, 50.2% of participants did physical activity 1–4x/month with their partner, 31.1% did with close family, 27.9% did with close friends (27.9%), and 13.9% did with neighbors. Across time, physical activity done 1–4x/month with close family showed the greatest decline, from 31.1% of participants at baseline to 20.8% 7 years later.

### Physical Activity Done with a Companion over Seven Years

#### Physical Activity with a Partner

The best fit model for physical activity done with a partner included the covariates of gender, education, and self-rated health (see Table 2). Compared to baseline, there were no significant differences in physical activity with a partner 1–4x/month after 2 years or 4 years, but participants were 25% less likely to do physical activity with a partner 1–4x/month after 7 years. Compared to baseline, participants were 30% less likely to do physical activity with a partner ≥5x/month after 2 years, 35% less likely after 4 years, and 29% less likely after 7 years.

**Table 2.** Associations Between Frequency of Physical Activity Done With a **Partner** and Time Over Seven Years.

Variable	Never versus 1–4x/month OR (95% CI)	Never versus ≥5x/month OR (95% CI)
Timepoint		
1 (2009)	1.00	1.00
1 vs. 2 (2011)	.84 (.70–1.00)	.70 (.56–.87)**
1 vs. 3 (2013)	.84 (.69–1.00)	.65 (.52–.81)***
1 vs. 4 (2016)	.75 (.62–.92)**	.71 (.56–.90)**
Gender		
Men	1.00	1.00
Women	.79 (.65–.92)*	.90 (.72–1.13)
Education		
Year 10 or less	1.00	1.00
Year 11 or 12	1.23 (.89–1.69)	1.56 (1.07–2.26)*
Certificate/Diploma	1.16 (.90–1.48)	1.18 (1.76–2.11)**
Bachelor’s degree or higher	1.53 (1.19–1.98)**	2.11 (1.57–2.85)***
Self-rated health		
Excellent/Very good	1.00	1.00
Good	.79 (.66–.94)**	.76 (.62–.94)*
Fair/Poor	.58 (.46–.73)***	.43 (.32–.58)***

*p < 0.05, **p < 0.01, ***p < 0.001.

Women were 21% less likely than men to do physical activity with a partner 1–4 x/month, but there were no gender differences between those who were physically active with a partner ≥5x/month. Participants with a university education were 53% more likely than those with less than grade 10 education to be physically active with a partner 1–4x/month and 111% more likely for ≥5x/month. Compared to those with a year 10 or less education, participants with years 11 or 12 education were 23% more likely to do physical activity with a partner 1–4x/month and 56% more likely to do activity ≥5x/month. People with fair/poor and good health were 42% less likely than those with very good/excellent health to do physical activity with a partner for either 1–4x/month and 57% less likely ≥5x/month.

#### Physical Activity with Close Family

The best fit model for physical activity done with close family included the covariates of living arrangement, gender, education, and self-rated health (see Table 3). There was a significant decrease in being physically active with close family for the 1–4x/month and ≥5x/month frequency. Compared to baseline, participants were 34% less likely to do physical activity with family 1–4 x/month after 2 years, 40% less likely after 4 years, and 45% less likely after 7 years. Compared to baseline, participants were 53% less likely to do physical activity with family ≥5x/month after 4 years, and 46% less likely after 7 years. There were no significant differences from baseline in physical activity with close family ≥5x/month after 2 years.

**Table 3.** Associations Between Frequency of Physical Activity Done With Close **Family** and Time Over Seven Years.

Variable	Never versus 1–4x/month OR (95% CI)	Never versus ≥5x/month OR (95% CI)
Timepoint		
1 (2009)	1.00	1.00
1 vs. 2 (2011)	.66 (.54–.80)***	.75 (.53–1.05)
1 vs. 3 (2013)	.60 (.49–.73)***	.47 (.32–.70)***
1 vs. 4 (2016)	.55 (.44–.68)***	.54 (.36–.80)**
Living arrangement		
Living alone	1.00	1.00
Living with others	1.56 (1.23–1.97)***	1.59 (1.09–2.34)*
Gender		
Men	1.00	1.00
Women	1.82 (1.47–2.26)***	1.72 (1.25–2.37)***
Education		
Year 10 or less	1.00	1.00
Year 11 or 12	1.24 (.88–1.74)	.66 (.37–1.18)
Certificate/Diploma	1.54 (1.18–2.00)**	1.03 (.70–1.53)
Bachelor’s degree or higher	1.69 (1.29–2.20)***	1.38 (.95–2.00)
Self-rated health		
Excellent/Very good	1.00	1.00
Good	.95 (.79–1.15)	.77 (.57–1.06)
Fair/Poor	.71 (.55–.92)**	.49 (.32–.76)**

*p < 0.05, **p < 0.01, ***p < 0.001.

Those living with others were 56% more likely than those who lived alone to do physical activity with close family 1–4x/month, and 59% more likely to do activity with close family ≥5x/month. Women were 82% more likely than men to do physical activity with close family for 1–4x/month, and 72% more likely to do activity with close family ≥5x/month.

Compared to those with a year 10 or less education, participants with a certificate/diploma education were 54% more likely to do physical activity with close family 1–4x/month, and those with a university degree were 69% more likely. There was no significant relationship between doing physical activity with close family ≥5x/month and educational attainment. People with fair/poor health were 29% less likely than those with very good/excellent health to do physical activity with close family 1–4x/month and 51% less likely to do activity with close family ≥5x/month.

#### Physical Activity with Close Friends

The best fit model for doing physical activity with close friends was the model with timepoint only (see Table 4). Compared to baseline, participants were 24% less likely to do physical activity with close friends 1–4x/month after 2 years, and 21% less likely after 7 years. In addition, there were no significant differences between baseline and 4 years later. There were no significant differences across time for doing physical activity with close friends ≥5x/month.

**Table 4.** Associations Between Frequency of Physical Activity With Close **Friends** and Time Over Seven Years.

Variable	Never versus 1–4x/month OR (95% CI)	Never versus ≥5x/month OR (95% CI)
Timepoint		
1 (2009)	1.00	1.00
1 vs. 2 (2011)	.76 (.62–.92) **	.99 (.71–1.27)
1 vs. 3 (2013)	.94 (.77–1.15)	1.15 (.83–1.58)
1 vs. 4 (2016)	.79 (.64–.98) *	1.23 (.88–1.71)

*p < 0.05, **p < 0.01, ***p < 0.001.

#### Physical Activity with Neighbors

The best fit model for doing physical activity with neighbors included the covariates of gender, education, and employment (see Table 5). Compared to baseline, participants were 29% less likely to do physical activity with neighbors 1–4x/month after 2 years, 34% less likely after 4 years, and 27% less likely after 7 years. Compared to baseline, participants were 41% more likely to do physical activity with neighbors ≥5x/month after 7 years, but there were no significant differences between baseline and 2 or 4 years later.

**Table 5.** Associations Between Frequency Physical Activity With **Neighbors** and Time Over Seven Years.

Variable	Never versus 1–4x/month OR (95% CI)	Never versus ≥5x/month OR (95% CI)
Timepoint		
1 (2009)	1.00	1.00
1 vs. 2 (2011)	.71 (.55–.92)**	1.41 (.91–2.17)
1 vs. 3 (2013)	.66 (.51–.86)**	1.29 (.83–2.02)
1 vs. 4 (2016)	.73 (.56–.97)**	1.59 (1.01–2.51)*
Gender		
Men	1.00	1.00
Women	1.44 (1.06–1.95)*	1.42 (.99–2.04)
Education		
Year 10 or less	1.00	1.00
Year 11 or 12	1.14 (.69–1.88)	1.25 (.71–2.19)
Certificate/Diploma	1.37 (.93–2.00)	1.09 (.69–1.71)
Bachelor’s degree or higher	1.77 (1.21–2.60) **	1.37 (.88–2.13)
Employment		
In paid employment	1.00	1.00
Not in paid employment	1.70 (1.21–2.25)***	1.18 (.80–1.72)

*p < 0.05, **p < 0.01, ***p < 0.001.

Women were 44% more likely than men to do physical activity with neighbors 1–4x/month, but there were no significant gender differences between those who were physically active with neighbors ≥5x/month. Participants with a university education were 77% more likely than those with less than grade 10 education to do physical activity with neighbors for 1–4x/month, and there was no significant relationship between education and doing physical activity with neighbors ≥5x/month. Those who were not in paid employment were 70% more likely to do physical activity with neighbors 1–4x/month than those who were not in paid employment, and there was no significant relationship between employment and doing physical activity with neighbors ≥5x/month.

## Discussion

The current study aimed to explore how often adults 60+ years did physical activity with companions and if this changed over 7 years. The most common companion was the participant’s partner, with just over half of the participants doing physical activity with their partner at least once per month at baseline. The least common companion was neighbors. Across time, there was a decrease in physical activity done with a partner and with close family. There was a decline between baseline and 7 years later for doing physical activity with close friends and with neighbors 1–4x/month, but not for ≥5x/month.

Partner was the most common companion, with 50% of the respondents reporting being physically active with their partner at least 1–4x/month at baseline. This is perhaps unsurprising due to the proximal access of partners. A previous study found that older adults had higher odds of meeting physical activity guidelines when their partner was also reaching guidelines, and changes in an individual’s physical activity was positively associated with changes in their partner’s physical activity (Cobb et al., 2015). In the current study, men were significantly more likely than women to do physical activity with a partner 1–4x/month. However, there were no significant gender differences for doing physical activity with a partner ≥5 ×/month. Previous research has reported that men are more likely than women to receive general social support from their partner, and the gender gap between received partner support increases with age (Schwarzer & Gutiérrez-Doña, 2005; Umberson et al., 1996). Doing physical activity with a partner was also more common among those with a university education. Older adults with higher educational attainment may have more resources available (e.g., financial, social) to engage in positive health behaviors (Zajacova & Lawrence, 2018). Previous research has also demonstrated that those with a university education are more likely to be active than those with lower levels of education (Bauman et al., 2012), and this pattern would seem to extend to activity with a partner.

Close family and friends were the next most common companion, with 31% of respondents at baseline doing physical activity at least 1–4x/month with close family and 28% doing physical activity with close friends. This is consistent with a cross-sectional study of older adults by Böhm et al. (2016), which found that family was a more common activity companion than friends. Previous studies have found that those who have the company of family and friends for physical activity are significantly more likely to reach physical activity guidelines than those who do not have such company (Böhm et al., 2016; Booth et al., 2000). Neighbors were the least common type of companion, with only 14% of respondents doing physical activity with neighbors at least 1–4x/month at baseline.

The current study demonstrated some specific gender differences in frequency of physical activity done with companions. Women were significantly more likely than men to do physical activity with close family members. However, there were no gender differences in doing physical activity with friends. Previous research has found that women report more social support from their adult children than men, whereas men report more social support from their partners than women (Lynch, 1998; Umberson et al., 1996). Women were also significantly more likely than men to do physical activity with neighbors. This is in line with previous research, which has found that women are significantly more likely than men to spend time with their neighbors (Campbell & Lee, 1990). Women may be more likely to draw on family members and neighbors as physical activity companions if their male partners are still working. In this current study and national population-based statistics for this age cohort (ABS, 2015), men 60+ years are more likely to be in the workforce than women 60+.

Frequency of doing physical activity with a close family member showed the greatest decline over time. After baseline, respondents were 35% less likely to do physical activity with a close family member 1–4x/month after 2 years, 40% less likely after 4 years, and 45% less likely after 7 years. After 7 years, respondents were 58% less likely to do physical activity with family ≥5x/month. Previous research which tracked social relationships in older adults but did not focus on physical activity, reported stable levels of contact with family across time (Shaw et al., 2007), which suggests that changes in family contact may not explain changes in frequency of activity done with family. If the family member is younger (e.g., an adult child), there could be a perceived difference in functional capability, and the older adult may not want to be a burden and so discontinue physical activity. Qualitative research has shown that not wanting to be a burden is a leading theme among older adults’ view of family involvement in care (Cahill et al., 2009). Alternatively, if the close family member is older (e.g., a sibling), declines in that person’s functioning may constrain physical activity participation and companionship. Family members may also have competing demands, for example, career progression and supporting children’s activities, which can limit time for physical activity with older adults.

There was also a reduction in frequency of doing physical activity with a partner. Seven years after baseline, respondents were 25% less likely to do physical activity with their partner 1–4x/month and 29% less likely to do it ≥5x/month. This may also be due to a functional decline from aging and a decline in physical activity generally. In line with this, a large synthesis of population studies found that, on average, there was a 3% increase in accumulative health-related problems every 12 months in adults 65+ (Mitnitski et al., 2005).

The frequency of doing physical activity with close friends and neighbors 1–4x/month declined between baseline and 7 years later, with participants 21% less likely to do physical activity with friends and 27% less likely to do physical activity with neighbors. However, it was interesting to note there was not a decline in frequency of physical activity with friends or neighbors ≥5x/times a month across time. There was a trend suggesting an *increase* in doing physical activity with friends and neighbors ≥5x/month, and this reached significance for activity done with neighbors 7 years after baseline. The contrast between 1–4x/month and ≥5x/month may be a result of those who are frequent exercisers maintaining their physical activity, whereas casual exercisers are more vulnerable to changes in behavior. In line with this, a population study looking at physical activity trajectories over 20 years in men aged 50–70 years identified three patterns of physical activity: low activity and decreasing (30% of participants), low activity but stable (46% of participants), and moderate activity but increasing (20.1% of participants) (Aggio et al., 2017). Friends and neighbor companionship for physical activity may be more stable as these people can be drawn from a wide variety of sources, for example, community groups, whereas partner and family companionship, although more convenient, is a finite resource vulnerable to decline.

The findings from this study contribute to the understanding of physical activity among older adults, as well as potential companionship for physical activity. Declines in frequency of physical activity done with partner and close family over time may reflect declines in physical activity generally among older adults. Several factors contribute to activity decline in older age, most notably poor health (McPhee et al., 2016). Alternatively, people may continue with physical activity but without a companion. Declines in physical activity done with a companion may also reflect declines in companionship availability. Older adults may be vulnerable to companionship disruptions due to life events such as loss of significant others and friends (Charles & Carstensen, 2010). Previous research has reported a 50% reduction in social network size between the ages of 50–59 and 60–69 years (Bruine de Bruin et al., 2020).

If declines in frequency of physical activity done with companions does reflect an increasing unavailability of companions, and adverse effects on physical activity level overall, then there are some implications for social interventions to promote physical activity maintenance among older adults. Ho et al. (2018) examined objectively measured social network size and found that after controlling for demographic, health, and accelerometry covariates, the larger the participants’ network size, the higher their levels of physical activity (*β* = 4.77, *p* = .042). Loprinzi and Joyner (2016) found that compared to those with no close friends, those with 1 close friend had a 1.7-fold increase in the odds of meeting physical activity guidelines and having 5 close friends produced a 2.71-fold increase in odds of reaching guidelines. Interventions could therefore focus on creating and broadening active social networks for older adults to promote physical activity engagement. Examples include buddy-systems (Thomas et al., 2012), peer-led interventions (Matz-Costa et al., 2018), community-based group interventions such as walking groups (Ball et al., 2017) and group-based activities (Bennett et al., 2018). Community group-based physical activity options have good adherence among older adults (Farrance et al., 2015).

If companionship is important for maintaining physical activity, general practitioners, health workers, and community support workers who promote physical activity among older adults could monitor available companionship. The results of the current study indicate that men who do not have a partner, and older adults living alone, in poor health, or with lower levels of education may be particularly vulnerable in terms of reduced frequency of physical activity with a companion over time. Older adults who rely on family for physical activity companionship and those who are active with a companion only 1–4x/month are also more vulnerable to declines in physical activity than those who are active with companions ≥5 ×/month. For those older adults interested in companionship for activity, providers may offer options such as local walking groups. Previous qualitative studies have found that lack of transport and affordability are key barriers to physical activity participation in older adults (Boulton et al., 2018; Franco et al., 2015), particularly in women (Moschny et al., 2011). Therefore, neighborhood companions for physical activity may be useful as they may not require transport, and costs for physical activity such as walking in local areas can be low. These opportunities should also reflect older adults’ interests: Older adults are more likely than mid-age adults to prefer physical activity done with people of the same age (Burton et al., 2012), and older women are more likely than men to prefer activities done with people of the same gender (van Uffelen et al., 2017). Campaigns promoting physical activity for older adults could highlight additional benefits of doing physical activity with a companion and consider options for family, friends, and partners to be physically active together.

To the best of our knowledge, this is the first prospective study to examine changes in frequency of physical activity done with companions among older adults across time. This study differentiated between friends, family, partners, and neighbors, enabling a more comprehensive understanding of activity companionship sources and vulnerability to declines over time. The large sample allowed us to control for covariates, including gender, education, living arrangements, employment status, and self-rated health.

This study has several limitations. Self-report data were used to assess activity frequency and are vulnerable to social desirability and recall bias. Data were derived from an overarching study of health and recreation, which may have attracted only those interested in this topic. A high proportion of participants in the source study, and the current study, were classified as meeting physical activity recommendations, and different results may have been obtained with a less active sample. Participants were located in one Australian capital city, and only young older adults were included. Therefore, the results may not generalize to other types of older adults such as older adults aged 75+, those living in rural areas, or non-Western populations. For example, prior research has found that people living in countries that have greater economic equality and are high in indulgence place more value on friendships than other countries (Lu et al., 2020). This might influence the companions people choose to be active with. Research has identified a lack of companionship for physical activity among older adults across a range of countries including Brazil (Böhm et al., 2016), Germany (Moschny et al., 2011), and Malaysia (Marthammuthu et al., 2021).

Some sources of physical activity companionship were not specified (e.g., personal trainers) in the measure used, and respondents may have interpreted the specific activity companions differently, for example, exercise class peers may have been considered as friends or not. The survey items assessing frequency of activity with the various companions included response options of “never” and “not applicable,” which respondents may have interpreted differently: A “never” response could indicate that participants may be active but without a companion or that the person was not physically active. Additionally, survey items did not ask what type of physical activity respondents were doing with companions. The proportion of participants who did physical activity with friends, family, or neighbors was small, which influenced the group sizes in the analyses and may have led to the statistical models being underpowered and therefore missing out on identifying some covariates.

More research is needed to understand what contributes to the decline in frequency of physical activity done with partner and close family companions over time, and why high frequency activity with friends and neighbors is maintained. Future research could also investigate what types of physical activity older adults are doing with companions, and if changes over time differ by activity type. Exploring other potential companionship sources such as exercise group peers, and physical activity supervisors may be important. In addition, future research could investigate if changes in companionship for physical activity are associated with changes in physical activity.

### Summary

Companionship has previously been identified as one of the most beneficial types of social support for physical activity in older adults. This study demonstrated that a partner was the most frequent companion for physical activity. From baseline to 7 years, there was a decrease in the frequency of physical activity done with a partner and a strong decrease in physical activity done with close family. Although there was a decline in physical activity 1–4x/month with close friends and neighbors, there was no decline for physical activity ≥5x/month. Men without a partner, and those living alone, in poor health, or with lower levels of education may be most vulnerable to reduced frequency of physical activity with companions. Further research is needed to understand the factors contributing to declines over time in physical activity done with a companion and the potential of social interventions to address this. At a population level, improved physical activity among older adults will increase wellbeing and reduce the burden of disease associated with inactivity and the related strain on government resources predicted with an aging population.

## Supplemental Material

Supplemental Material - Frequency of Physical Activity Done with a Companion: Changes Over Seven Years in Adults Aged 60+ Living in an Australian Capital City Click here for additional data file. Supplemental Material for Frequency of Physical Activity Done with a Companion: Changes Over Seven Years in Adults Aged 60+ Living in an Australian Capital City by Genevieve S. E. Smith, MAppPsych, Wendy Moyle, PhD, and Nicola W. Burton, PhD in Journal of Aging and Health
