# Supplementary material for: Frequency of Physical Activity Done with a Companion: Changes Over Seven Years in Adults Aged 60+ Living in an Australian Capital City
Source: J Aging Health. 2023 Feb 28;35(9):736–48. doi: 10.1177/08982643231158424 (PMC10478325; doi:10.1177/08982643231158424)
Supplement: Supplemental Material - Frequency of Physical Activity Done with a Companion: Changes Over Seven Years in Adults Aged 60+ Living in an Australian Capital City [file sj-pdf-1-jah-10.1177_08982643231158424.pdf]

Supplementary materials

Table 1s

Akaike Information Criterion (AIC) values for Partner models

Model	Fixed effects	Random Effect	df	AIC
**1**	wave	participant	11	8855
**2**	wave + sex	participant	13	8724
**3**	wave+ sex + health rating	participant	17	8705
**4**	**wave + sex + health rating + education**	**participant**	**23**	**8690**
**5**	wave + sex + health rating + education + employment	participant	25	8708
**6**	wave + health rating + education+ employment	participant	23	8793

Note: A lower AIC value indicates better quality of fit relative to the complexity of the model; therefore, the model with the lowest AIC was selected

Table 2s

Akaike Information Criterion (AIC) values for Family models

Model	Fixed effects	Random Effect	df	AIC
**1**	wave	participant	11	6209
**2**	wave + sex	participant	13	6174
**3**	wave + sex + living arrangement	participant	15	6140
**4**	wave+ sex + living arrangement + health rating	participant	19	6120
**5**	**wave+ sex + living arrangement + health rating + education**	**participant**	**25**	**6105**
**6**	wave+ sex + living arrangement + health rating + education + employment	participant	27	6106

Note: A lower AIC value indicates better quality of fit relative to the complexity of the model; therefore, the model with the lowest AIC was selected

Table 3s

Akaike Information Criterion (AIC) values for Friend models

Model	Fixed effects	Random Effect	df	AIC
**1**	**wave**	**participant**	**11**	**6416**
**2**	wave + sex	participant	13	6706
**3**	wave + living arrangement	participant	13	6849
**4**	wave + education	participant	17	6924
**5**	wave + employment	participant	13	6433
**6**	wave + health rating	participant	15	6734

Note: A lower AIC value indicates better quality of fit relative to the complexity of the model; therefore, the model with the lowest AIC was selected

Table 4s

Akaike Information Criterion (AIC) values for Neighbor models

Model	Fixed effects	Random Effect	df	AIC
**1**	wave	participant	11	3529
**2**	wave + sex	participant	13	3529
**3**	wave + sex + living arrangement	participant	15	3537
**4**	wave + sex + education	participant	19	3505
**5**	wave + education	participant	17	3514
**6**	**wave + sex + education + employment**	**participant**	**21**	**3478**
**7**	Wave + sex + education + employment + health rating	participant	23	3487

Note: A lower AIC value indicates better quality of fit relative to the complexity of the model; therefore, the model with the lowest AIC was selected
